# Exploration of key drug target proteins highlighting their related regulatory molecules, functional pathways and drug candidates associated with delirium: evidence from meta-data analyses

**DOI:** 10.1186/s12877-023-04457-1

**Published:** 2023-11-22

**Authors:** Md Parvez Mosharaf, Khorshed Alam, Jeff Gow, Rashidul Alam Mahumud

**Affiliations:** 1 School of Business, Faculty of Business, Education, Law and Arts, University of Southern Queensland, Toowoomba, QLD 4350 Australia; 2 Bioinformatics Lab, Department of Statistics, University of Rajshahi, Rajshahi, 6205 Bangladesh; 3 School of Accounting, Economics and Finance, University of KwaZulu-Natal, Durban, 4000 South Africa; 4 NHMRC Clinical Trials Centre, Faculty of Medicine and Health, The University of Sydney, Camperdown, NSW 2006 Australia

**Keywords:** Delirium, Hub-proteins, Transcription factors, microRNA, Drug repurposing

## Abstract

**Background:**

Delirium is a prevalent neuropsychiatric medical phenomenon that causes serious emergency outcomes, including mortality and morbidity. It also increases the suffering and the economic burden for families and carers. Unfortunately, the pathophysiology of delirium is still unknown, which is a major obstacle to therapeutic development. The modern network-based system biology and multi-omics analysis approach has been widely used to recover the key drug target biomolecules and signaling pathways associated with disease pathophysiology. This study aimed to identify the major drug target hub-proteins associated with delirium, their regulatory molecules with functional pathways, and repurposable drug candidates for delirium treatment.

**Methods:**

We used a comprehensive proteomic seed dataset derived from a systematic literature review and the Comparative Toxicogenomics Database (CTD). An integrated multi-omics network-based bioinformatics approach was utilized in this study. The STRING database was used to construct the protein-protein interaction (PPI) network. The gene set enrichment and signaling pathways analysis, the regulatory transcription factors and microRNAs were conducted using delirium-associated genes. Finally, hub-proteins associated repurposable drugs were retrieved from CMap database.

**Results:**

We have distinguished 11 drug targeted hub-proteins (MAPK1, MAPK3, TP53, JUN, STAT3, SRC, RELA, AKT1, MAPK14, HSP90AA1 and DLG4), 5 transcription factors (FOXC1, GATA2, YY1, TFAP2A and SREBF1) and 6 microRNA (miR-375, miR-17-5, miR-17-5p, miR-106a-5p, miR-125b-5p, and miR-125a-5p) associated with delirium. The functional enrichment and pathway analysis revealed the cytokines, inflammation, postoperative pain, oxidative stress-associated pathways, developmental biology, shigellosis and cellular senescence which are closely connected with delirium development and the hallmarks of aging. The hub-proteins associated computationally identified repurposable drugs were retrieved from database. The predicted drug molecules including aspirin, irbesartan, ephedrine-(racemic), nedocromil, and guanidine were characterized as anti-inflammatory, stimulating the central nervous system, neuroprotective medication based on the existing literatures. The drug molecules may play an important role for therapeutic development against delirium if they are investigated more extensively through clinical trials and various wet lab experiments.

**Conclusion:**

This study could possibly help future research on investigating the delirium-associated therapeutic target biomarker hub-proteins and repurposed drug compounds. These results will also aid understanding of the molecular mechanisms that underlie the pathophysiology of delirium onset and molecular function.

**Supplementary Information:**

The online version contains supplementary material available at 10.1186/s12877-023-04457-1.

## Background

Delirium is a serious neuropsychiatric medical condition triggered by multiple predisposing and precipitating factors, including critical medical situations, drug usage or withdrawal, and major surgery [1, 2]. It is a common phenomenon that increases mortality and morbidity, and prolongs hospital stays, and increases overall costs [3, 4]. Delirium causes a great deal of suffering in both carers and patients [2, 5]. More than 50% of delirious cases are undiagnosed in hospitalized patients, particularly in intensive care units (ICU) [4, 6–9]. The underlying causes for the difficulties of diagnosis reveal the deficiency of definition about delirium and ICU syndrome, other associated factors, and most importantly, the entire molecular pathophysiology of delirium [10]. The entire disease pathophysiology consists of risk markers, disease markers, end products and their combination acting in different biological processes for disease development and proliferation. The current literature suggested that various factors related to the pathophysiology of delirium have been studied, including inflammation and neuroinflammation, chronic stress, impaired blood brain barrier integrity, neuronal injury, reduced neuroprotection, cholinergic deficiency, and the effects of anticholinergic drug [11]. The pathophysiology of delirium may be understood by identifying biomarkers, molecular pathways, and predictors of delirium using multi-omics-based system biology techniques.

Delirium is prompted by distinct factors, however, the pathophysiological mechanism of delirium presentation is still unknown [2, 12, 13]. Molecular key components also play a major and significant role in delirium development, with various studies revealing the risk factors, including the genetic causes, proteins, and other biomolecules in its appearance [4, 14–18]. The few genetic investigations that have been conducted are powered modestly, and hence, no consistent genomic or proteomic signature candidate linked to delirium risk, diagnosis, and therapeutics have been found [1, 19, 20]. Therefore, the entire pathophysiology related to delirium has remained unknown, thus demanding an in-depth rigorous molecular investigation.

However, significant molecular biomarkers can act as risk and disease markers [21]. Modern transcriptional and proteomic data analysis provides phenomenal insight into the key molecular biomarkers and their functional pathways for specific biological traits [22–26]. Furthermore, integrative network-based system biology and bioinformatics approaches are widely used for molecular investigation, illuminating mysterious disease pathogenesis. In this regard, significant proteomic biomarkers effectively reveal disease severity, risk, onset, recovery, and pathway of the illness.

A recent study conducted by Takahaschi et al. 2020 [27] reported some key delirium-associated genes and their associated functional pathways using a dataset from the Comparative Toxicogenomics Database (CTD). Although the study reported some important molecular insights, it exclusively used database-derived genomic data, with the exception of one gene expression dataset. The study failed to account for the crucial pre- and post-transcriptional regulatory molecules which are very important for controlling gene expression. The study also did not provide any information about the therapeutic agents associated with delirium.

Therefore, we focused on collecting a comprehensive seed genomic dataset including available gene expression data along with the database information. This study was designed to identify the delirium-associated key proteomic biomarkers, their associated pre- and post-transcriptional regulatory molecules, and their functional pathways using an integrative bioinformatics analytical approach. Using drug repurposing techniques, the probable computational repurposable drug candidates associated with the key proteomic biomarkers were also identified. The outcome of this study will serve as a rational basis for further molecular in-depth investigation regarding the key proteomic biomarkers of delirium focusing on diagnostic and therapeutic development.

## Materials and methods

### Data collection

This study constructed a comprehensive proteomic dataset by combining two data sources. Firstly, a thorough review of the literature was conducted on delirium-related proteomic signatures. The electronic bibliographic databases PubMed, Scopus, and EBSCOhost (CINAHL, Medline) were utilized and searched articles between January 1, 2000, and December 31, 2022. The primary keywords were “delirium” and “biomarker” used along with a combination of other associated keywords including “marker”, ‘genetic”, “proteomic”, “genes” and “protein” to search the studies. Boolean operators “AND”, and “OR” were applied to combine the searching keywords. Fifty-four studies were included in our analysis out of a total of 2,065 that were retrieved and reviewed. The delirium-associated unique proteomic signatures from those selected studies were collected, which included 154 unique gene-encoded proteins. The detailed procedure of the literature review for this data collection has been described in Supplementary File 1.

Second, delirium-associated genes were collected from the CTD (http://ctdbase.org/) [28] which has been widely used to explore the chemical-genes/proteins interactions, gene-disease interactions as well as chemical-disease interactions. These database-retrieved interactions help researchers better understand the disease mechanism brought on by chemicals or chemical-associated genes/proteins. Delirium-related genomic data were retrieved from the CTD using the term “delirium” as the search term. The CTD inference score > 40 was considered in this study to select the top-ranking delirium-associated genes [27]. A total of 350 delirium-related genes were extracted from CTD.

**Fig. 1:**
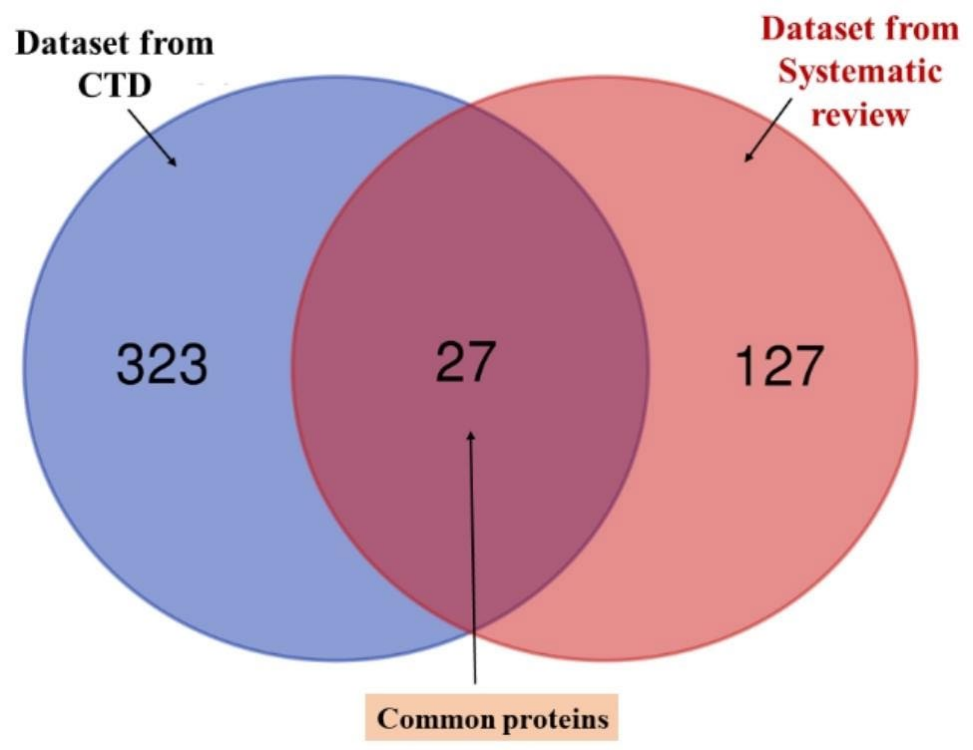
Venn diagram visualizing the seed dataset of this study

The final seed dataset for this study was constructed by combining the above two datasets. If the literature-retrieved dataset is noted by *A* and the CTD-retrieved dataset is annotated by *B*, then the ultimate dataset is Z=*(A*U*B).* The following Venn diagram describes the seed dataset distribution (Fig. 1).

### Methods

**Fig. 2:**
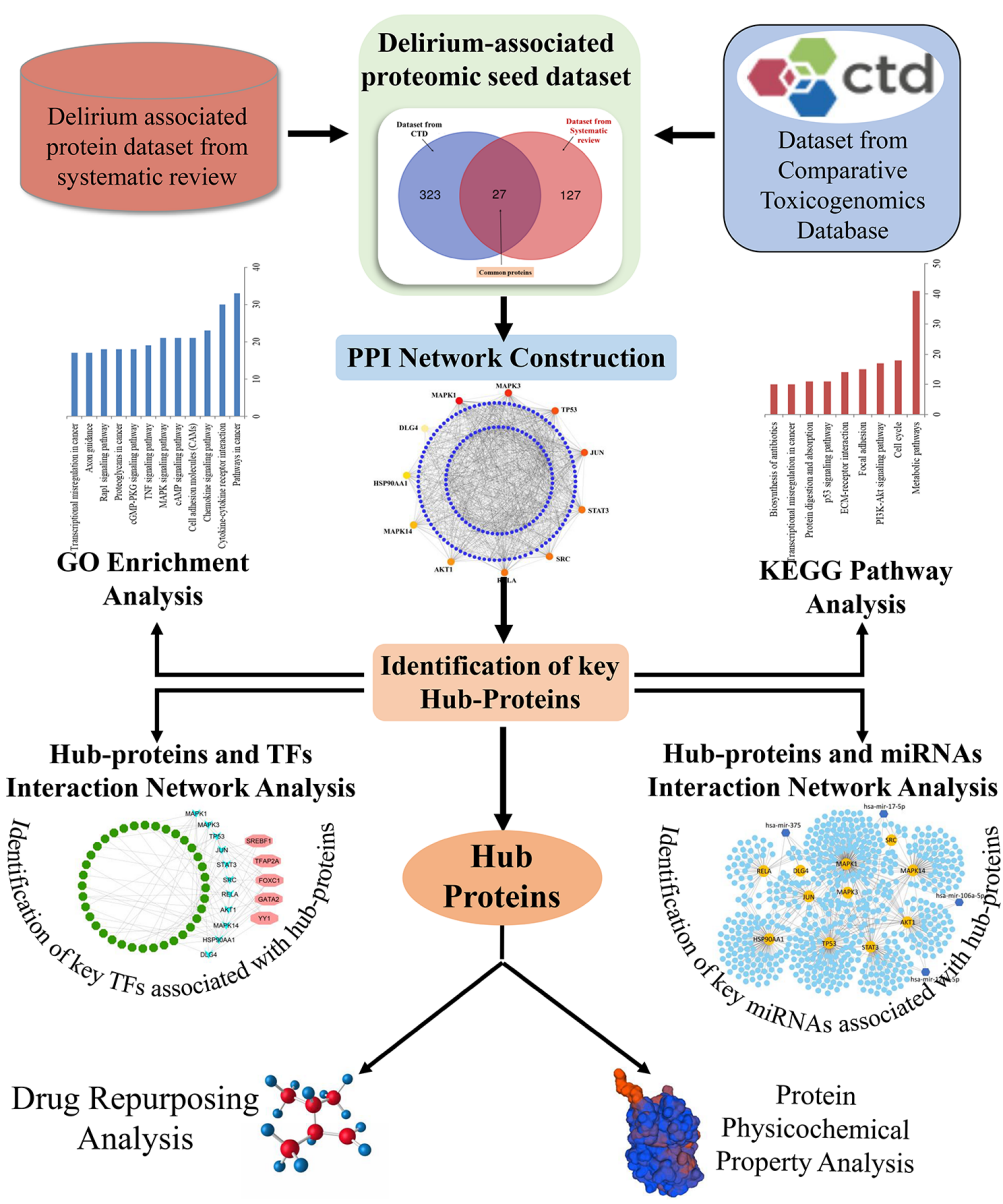
The global working flow diagram of this study

The widely used integrated bioinformatics and system biology analytic methodologies were applied in this study and are explained below. The transcriptome-guided network-based investigation employed a novel analytical technique to determine the leading biomolecules. The entire working flow diagram of this study is presented in Fig. 2.

#### Human protein-protein Interaction (PPI) network construction

To construct the human Protein-Protein Interaction (PPI) network, the seed dataset was utilized where the genes were mapped to the human proteins as gene-encoded proteins. To carry out a certain function, bigger protein complexes are formed with the help of the PPI network [29]. The top-ranked hub-proteins are considered the most significant proteins for biological function based on the number of connections with other nodes. PPI network analysis is one of the most effective and widely used techniques to reveal hub-proteins. In this study, the STRING database [30] has been used to construct the PPI network. PPI network topological studies and visualization were carried out using NetworkAnalyst [31] and Cytoscape 3.7.2 [31]. A topological exploration based on dual-metric measurements degree of connectivity and betweenness was utilized to identify the highly significant hub-proteins.

#### Physicochemical properties of hub proteins

The online project ProtParam (https://web.expasy.org/protparam/) provided the physicochemical characteristics of the hub-proteins that have been identified in this study. The server calculates numerous chemical and physical characteristics for a certain protein. For the reported hub protein, the molecular weight, theoretical pI, extinction coefficient, instability index, aliphatic index, and grand average of hydropathicity (GRAVY) were all examined in this study.

#### Functional and pathway enrichment analysis

The gene set enrichment and annotation analyses [32–35] which are known as the biological processes (BP), molecular functions (MF), and cellular components (CC) and the functional and signaling pathways were carried out using the top-ranked hub-proteins. The g:GOSt software embedded in the g:Profiler web server was utilized to perform the enrichment and annotation analyses. The signaling pathways related to delirium-associated key genes were retrieved from three different databases called, KEGG, REACTOME and WIKIPATHWAYS. The statistically significant gene ontology (GO) terms and the significant pathways for hub-proteins were defined by the adjusted p-values < 0.05 and the Benjamini and Hochberg [36] procedure controlling the false discovery rate ( FDR).

#### Regulatory network analysis

The significant pre- and post-transcriptional regulatory molecules related to delirium-associated proteins were identified by analyzing the hub-proteins and transcription factor (TF) interaction network as well as the hub-proteins and microRNA (miRNA) interaction network, respectively. For this purpose, the JASPAR [37] TF database was utilized to construct the TF-Hub-proteins interaction network. The TarBase V8.0 and miRTarBase [38, 39] miRNAs databases were utilized to construct the interaction networks among the hub-proteins and miRNAs. The key important regulatory molecules were selected using the NetworkAnalyst online server, which applied the maximum topological matrices (degree of interconnection and betweenness) to the interaction network.

#### Drug-repurposing analysis for the hub-proteins

The delirium-associated hub-proteins were used to identify the repurposable drugs/drug candidates. The online drug-repositioning tool and database Connectivity Map (CMap) were used to obtain the compounds that were likely to be medications or drug candidates based on delirium-associated hub-proteins guidance [49]. This platform integrates knowledge concerning drugs or drug candidate molecules from publicly accessible sources of data in clinical experimental phases, investigative stages, and stages where they have been authorized for use in treating patients. In this study, only FDA authorized and launched drugs that are related to the delirium-associated hub-proteins were collected.

## Results

### Dataset description

The comprehensive literature review of delirium-associated gene-encoded proteins was collected from the finally included studies. Finally, fifty-four studies revealed a set of 154 unique proteins. In the review, the included studies were conducted in different settings and in patients with critical medical conditions. The main goal was to identify the delirium-associated genomic/proteomic signature. The well-established and widely used delirium assessment methods were used to confirm the patient’s delirious condition. To collect the gene-encoded protein dataset, we have only considered delirium-associated genes and proteins. On the other side, the CTD database was utilized to retrieve the delirium-associated genomic signatures. The database provided a search outcome of 25,036 delirium-associated genes when the lower inference score was included. After setting an inference score cutoff (> 40), the number of genes decreased to 350 genes. The ultimate seed dataset was constructed by combining the two datasets from different sources, consisting of 477 unique gene-encoded proteins (Supplementary file 2). Between the two datasets, 323 unique proteins come from set A and 127 come from set B and 27 genes were found to be in common. Therefore, for the downstream analysis, 477 genes/proteins were utilized in this study.

### PPI network analysis

**Fig. 3:**
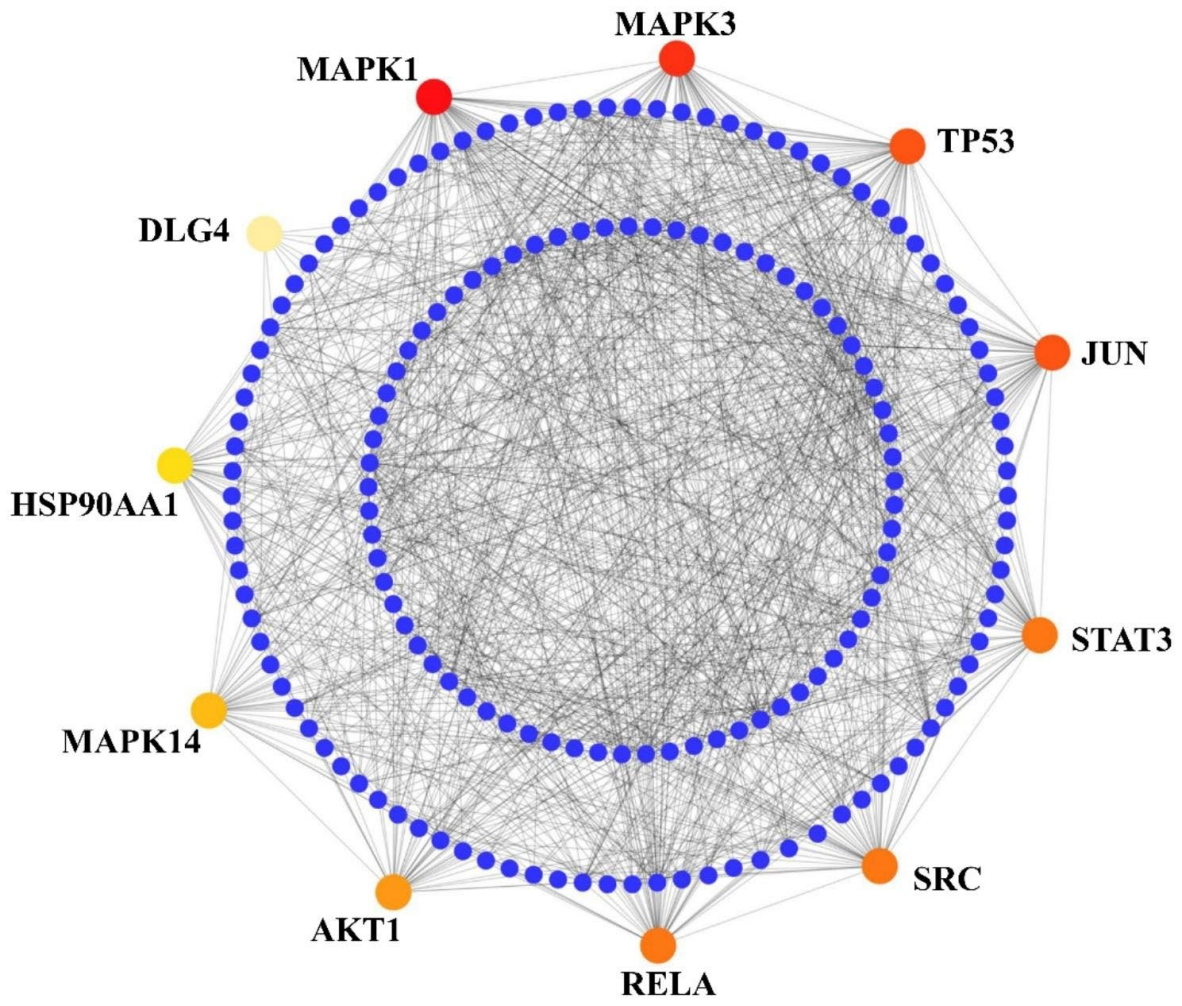
PPI network of delirium-associated proteins. The smaller dark blue circular nodes represent the network proteins. The bigger circular colorful nodes represent the hub-proteins where a redder color node means a higher degree of connectivity and a more yellow color node means a lower degree of connectivity measured by CytoHubba

The PPI network of the 477 collected gene encoded proteins, was constructed using the STRING database (Fig. 3). Among the 477 proteins, 4 proteins were not detected by STRING database, hence they were excluded from the network. A dual-metric topological measurement of the degree of connectivity and the betweenness were considered to identify the central highly connected representative hub-proteins. The key 11 hub-proteins were identified based on their degree and betweenness using topological analysis measured by the CytoHubba. The hub-proteins are MAPK1, MAPK3, TP53, JUN, STAT3, SRC, RELA, AKT1, MAPK14, HSP90AA1 and DLG4. The hub-proteins are highlighted in Fig. 3. These hub-proteins were different compared to those in the previously conducted study by Takahaschi et al. 2020 [27].

This study reports the physicochemical characteristics of the detected delirium-associated hub-proteins. The physicochemical properties are necessary for a more thorough analysis of the reported key biomolecules. The number of amino acids varied from 331 (JUN) to 770 (STAT3) among the hub-proteins when they also have the lowest (35675.57 kda) and highest (88067.80 kda) molecular weight, respectively. The theoretical isoelectric point (pI) revealed that the hub-protein JUN consisted of the highest pI value of 8.90 and the lowest pI value of 4.94 was contained in the HSP90AA1 hub-protein. Detailed properties information of the hub-proteins is provided in Table 1.

**Table 1:** The physicochemical properties of the reported hub-proteins

Hub Protein’s Name	# Of amino acids	Molecular weight (kda)	Theoretical pI	# of negatively charged residues (Asp + Glu)	# of positively charged residues (Arg + Lys)	*Extinction coefficient	Instability index	Aliphatic index	Grand average of hydropathicity (GRAVY)
MAPK1	360	41389.71	6.50	45	42	45,185	39.71	95.94	-0.287
MAPK3	382	42986.99	6.87	48	47	47,370	50.66	74.03	-0.581
TP53	393	43653.18	6.33	50	46	36,035	73.59	59.08	-0.756
JUN	331	35675.57	8.90	28	32	7575	53.28	74.65	-0.469
STAT3	770	88067.80	5.94	91	82	114,665	48.22	83.45	-0.403
SRC	536	59834.76	7.10	63	63	84,270	43.76	71.74	-0.473
RELA	551	60219.18	5.46	65	54	23,880	54.44	73.16	-0.464
AKT1	480	55686.42	5.75	77	66	65,695	35.47	71.69	-0.575
MAPK14	360	41293.29	5.48	50	38	50,100	43.64	95.08	-0.261
HSP90AA1	732	84659.71	4.94	151	110	59,625	41.94	79.37	-0.750
DLG4	724	80495.37	5.58	108	86	66,615	45.67	83.38	-0.469

Note: *Extinction coefficients are in units of M^− 1^ cm^− 1^, at 280 nm measured in water

### Functional annotation and enrichment analysis

**Fig. 4:**
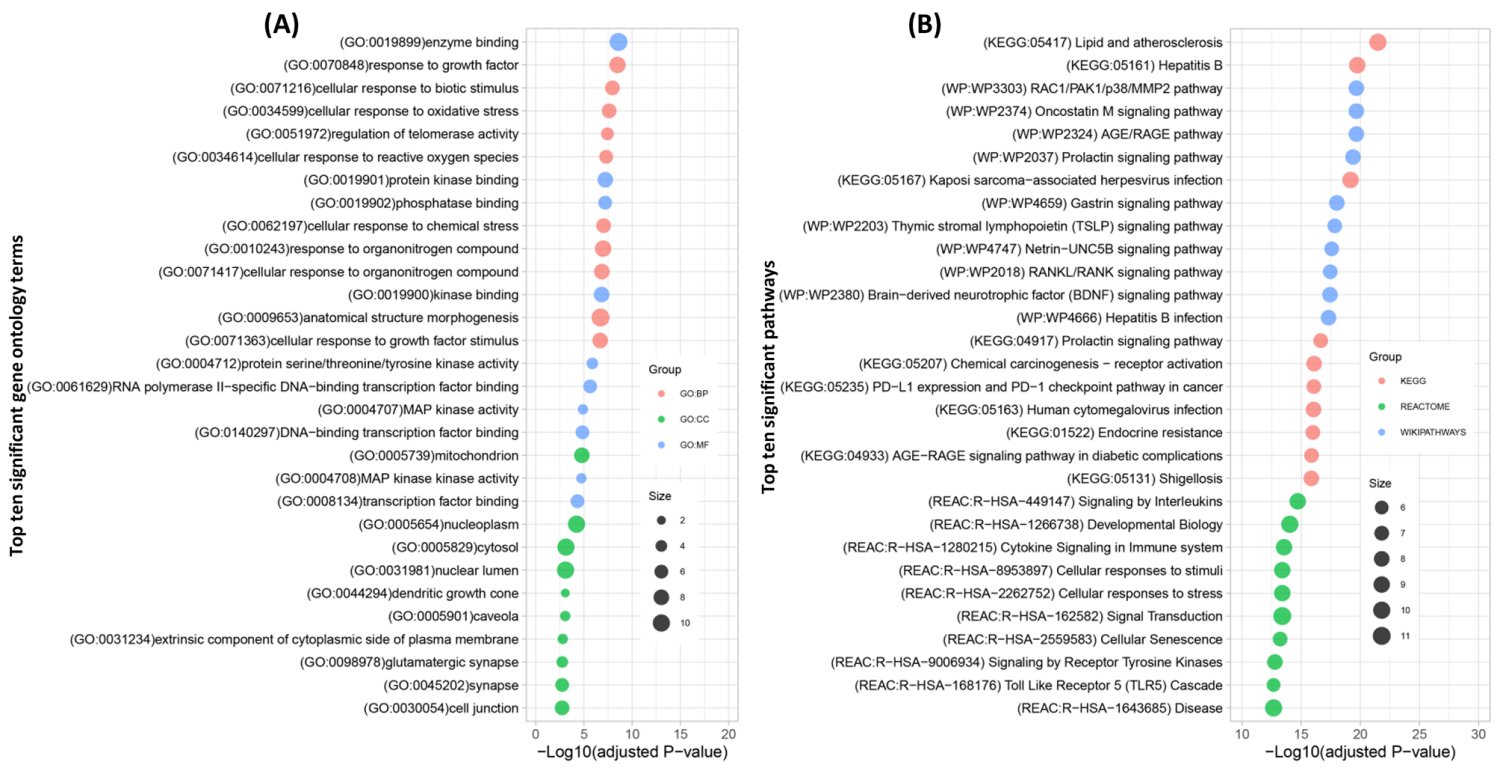
(**A**) The top ten significant GO terms associated with the biological process (BP), molecular function (MF) and cellular component (CC) has been presented. (**B**)The three-database retrieved top significant signaling pathways shared by delirium-associated key genes have been presented. In both figures, the size of the bubbles indicates the number of key genes enriched in each term

The gene ontology (GO) functional enrichment and annotation analysis of the hub-proteins are represented in three categories, namely the BP, MF, and CC. In Fig. 4A, the top ten significant and enriched GO terms from each of the three categories (BP, MF, and CC) have been summarized. The top ten functional signaling pathways of the hub-proteins from three different databases are reported in Fig. 4B. The GO functional enrichment and pathway analysis of the delirium-associated hub-proteins revealed a wide range of biological and functional pathways. Among the significant BPs, the response to organonitrogen compound, response to growth factor, cellular response to biotic stimulus, response to oxidative stress, cellular response to reactive oxygen species, cellular response to chemical stress, and regulation of signal transduction were highly considerable. The highly enriched and significant MFs were enzyme binding, protein kinase binding, phosphatase binding, kinase binding, and protein serine/threonine/tyrosine kinase activity. Moreover, glutamatergic synapse, nucleoplasm, cytosol, synapse, and nuclear lumen were the hub-proteins’ highly enriched and significant cellular locations (Fig. 4A).

On the other hand, the noticeable significant signaling pathways of the hub-proteins mainly consisted of receptor activities, stress and immune responses, cytokines and inflammation, and signaling and developmental pathways. The signaling pathways were those that Takahaschi et al. 2020 [27] identified in relation to delirium. The significant functional pathways shared by the hub-proteins were lipid and atherosclerosis, cell signal transduction, RAC1/PAK1/p38/MMP2 pathway, AGE/RAGE pathway, cytokine signaling pathway, developmental biology, shigellosis, cellular senescence, signaling by interleukins and other receptor associated pathways (Fig. 4B). The significantly enriched pathways are associated with the components of hallmarks of aging [40]. The details of functional enrichment and pathway analysis results are provided in Supplementary File 3.

### Identification of hub-proteins-related regulatory factors

**Fig. 5:**
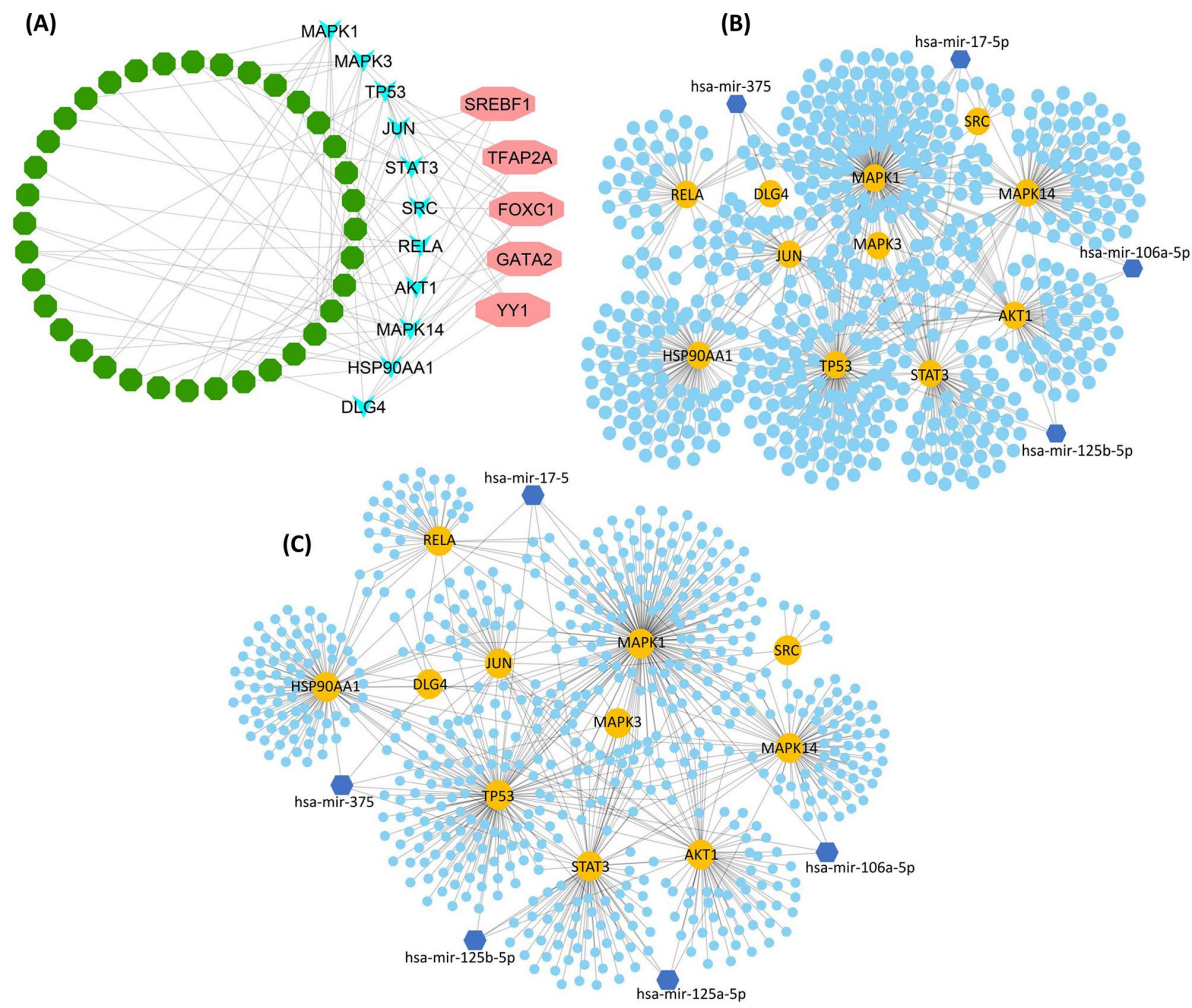
(**A**) TFs and the hub-proteins interaction network were constructed from the JASPAR database The pink-colored octagonal nodes represent the key TFs while the blue color triangle nodes are for hub-proteins and the green color nodes stand for the associated TFs. The miRNAs versus hub-proteins interaction network (**B**) were constructed from the TarBase, whereas the second network (**C**) was built using the miTarBase database. In both networks, the dark, blue-colored hexagonal nodes represent the key miRNAs. The yellow-colored circular nodes stand for hub-proteins and the light, blue-colored circular nodes represent the other associated miRNAs

The pre- and post-transcriptional regulatory molecules of the reported therapeutic target, hub-proteins, have been identified throughout the interaction network analysis. The interaction network among the drug-targeted proteins revealed the key TFs were, namely, FOXC1, GATA2, YY1, TFAP2A, and SREBF1 based on the topological analysis by CytoHubba (Fig. 5A). Similar topological measurements of degree and betweenness centrality were applied to the interaction network of miRNAs and hub proteins. The network analysis from the two databases showed similar results for the post-transcriptional regulatory miRNAs. The substantial top regulatory miRNAs were recorded as miR-375, miR-17-5, miR-17-5p, miR-106a-5p, miR-125b-5p, and miR-125a-5p (Fig. 5B C). Consistency among regulatory elements from different databases revealed the significance of these molecules for hub proteins in delirium.

### Drug-repurposing

The integrative comprehensive drug database CMap revealed a total of 17 FDA approved drug molecules targeting our proposed delirium-associated hub-proteins. Among the retrieved drugs, 11 of them are inhibitors, four were receptor drugs, one is a stimulant, and one is a mucolytic agent (Table 2). The only hub-protein, MAPK14 did not retrieve any associated repurposable drugs from the CMap database. Although all the hub-proteins also found many other repurposable drugs that are still in different clinical trial stages. However, these were ignored and not reported.

**Table 2:** The repurposed drug agents associated with the hub-proteins of delirium

Target proteins	Name of Drugs	Mechanism of Action	Disease area
MAPK1	Regorafenib	Inhibitor	Oncology
	Arsenic-trioxide	Stimulant	Gastroenterology
MAPK3	Arsenic-trioxide	Stimulant	Gastroenterology
TP53	Carbendazim	Inhibitor	Infectious disease
	Aspirin	Inhibitor	Neurology/psychiatry, endocrinology, dental
JUN	Vinblastine	Inhibitor	Hematologic malignancy, infectious disease, oncology
	Irbesartan	Receptor	Cardiology, nephrology
	Ephedrine-(racemic)	Receptor	Cardiology, pulmonary, neurology/psychiatry, endocrinology
	Arsenic-trioxide	Stimulant	Gastroenterology
STAT3	Niclosamide	Inhibitor	Infectious disease
	Acitretin	Receptor	Dermatology
SRC	Vandetanib	Inhibitor	Oncology
	Ponatinib	Inhibitor	Hematologic malignancy
	Dasatinib	Inhibitor	Hematologic malignancy
	Bosutinib	Inhibitor	Hematologic malignancy
RELA	Bortezomib	Inhibitor	hematologic malignancy
	Acetylcysteine	Mucolytic agent	Gastroenterology
AKT1	Arsenic-trioxide	Stimulant	Gastroenterology
HSP90AA1	Nedocromil	Receptor	Neurology/psychiatry, ophthalmology
DLG4	Guanidine	Inhibitor	Neurology/psychiatry

The repurposable drugs have shown effectiveness across different disease areas, including neurology/psychiatry, nephrology, cardiology, gastroenterology, various oncology, and others (Table 2). These computationally predicted drugs need deeper clinical investigation before serving as a potential treatment source for delirium.

## Discussion

The literature review showed that very few proteomic-level studies have been conducted to identify the key proteins associated with delirium [1, 19, 20]. Nevertheless, to the best of our knowledge, we were unable to locate any gene expression data regarding delirium. Due to the paucity of gene expression transcriptomics data, this study planned to collect a distinct molecular dataset focusing on the genes and proteins associated with delirium. Accordingly, a comprehensive systematic review of 2,065 articles revealed 54 studies that reported 154 unique delirium-associated gene-encoded proteins, although none of them were consistent across studies. On the other side, the CTD database provided a list of 350 gene-encoded proteins which assisted to make a comprehensive proteomic seed dataset of 477 unique gene-encoded proteins that could be analyzed. The current seed dataset contains more delirium-associated genomic indicators which were reported by high throughput gene expression data analysis under few targeted genes/proteins in different studies. This dataset diversity and its settings difference can explain the delirium-associated genes from different biological aspects which is highly significant and thus distinguishes this study from others. Based on this dataset, the current study aimed to identify the key proteomic biomarkers, their regulatory molecules, the functional pathways, and the repurposable drug components associated with delirium-associated hub-proteins. Using the network-based multi-omics data integration framework analysis approach, the study was able to identify the delirium-associated key pre-clinical substantial drug target biomolecules. This study outcome focused on highlighting the pathophysiology of delirium focused on the molecular functionality aspects.

The integrated bioinformatics analysis approach revealed 11 hub-proteins (MAPK1, MAPK3, TP53, JUN, STAT3, SRC, RELA, AKT1, MAPK14, HSP90AA1 and DLG4), 5 TFs (FOXC1, GATA2, YY1, TFAP2A and SREBF1) and 6 miRNAs (miR-375, miR-17-5, miR-17-5p, miR-106a-5p, miR-125b-5p, and miR-125a-5p) associated with delirium. The PPI network revealed that the hub-proteins differed from those of the other study [27] because of data diversity and study settings. This analytical dataset contained genes collected from different studies which were in distinct settings using biological samples of the human body. The system biology approach has the privilege of accumulating genomic information through the network-based analysis which reflects the global key hub-proteins for a specific disease. Among the hub-proteins, the mitogen-activated protein kinase 1 (MAPK1) proteins and its mutants are associated with neurodevelopmental disorders, which eventually phenotype with several neurological challenges including intellectual disability, growing delay, and interactive difficulties such as anxiety, reduced stress tolerance and aggressive behaviour [41]. In addition to pro-inflammatory stimuli, cellular and environmental stressors also activate the MAPK signaling pathways [42, 43], whereas delirious conditions are also triggered by the stressed medical condition and inflammatory cytokines activities [4, 44, 45]. The reported MAPK signaling pathway-related hub proteins, namely MAPK1, MAPK3 and MAPK14, might be involved in delirium-associated neurological disorders/activities which demand deeper investigation. The tumor suppressor gene, TP53 encoded proteins, is also known as TP53/p53, is a crucial protein in various cancer and tumor development [46–48]. A wide interaction with cytokine and chemokines inflammatory components is revealed by the p53 pathway and other family members [49], indicating a positive association with delirium development under critical and stressful medical conditions. The c-Jun type gene encoded proteins JUN has a greater involvement in different cell activities, disease proliferation, and stress-response signaling pathways [50–52]. The ischemic/reperfusion consequences in the perioperative period and the postoperative pain sedation are activated by the JAK/STAT3 signaling pathway with the assistance of other inflammatory cytokines (like, IL6). The research revealed that postoperative pain and inflammation are major causes of delirium [49–51], indicating a strong association of the above proteins with delirium phases. The SRC protein is crucial in chronic inflammation and cancer development [53]. The RELA protein is treated as a potential cancer biomarker, responsible for cytokine production and inflammatory bowel disease [54, 55], when AKT1 is a protein that aids in the correct growth and operation of the nervous system [56–58]. The HSP90AA1 gene-encoded protein’s expression is associated with the inflammatory protein interleukins (IL) and the regulatory role in neurodevelopment, indicating a core connection with cognitive dysfunction and delirium [59, 60]. The hub protein DLG4 is involved in the microglial inflammatory process, and genetic differences in DLG4 are linked to anatomical variations in the preterm newborn brain [61]. The above hub-proteins showed a diverse functionality related to delirium, other neural dysfunction and cognitive impairment. The linkage clearly indicates the significance of the hub-proteins in delirium-associated functional pathways and pathophysiology. Most of them revealed cytokines inflammation, neurological disorders/activities, stress responsiveness, and pain management, which are parallel causes of delirium in acute medical conditions.

Functional enrichment and signaling pathways analysis is one of the most effective ways to decipher a group of genes’ molecular activities and functional pathways. Among the GO terms, the cellular response to oxidative stress is an important pathway associated with chronic inflammation and aged diseases [62] which is enriched by seven hub-proteins. Oxidative stress might be associated with delirium pathophysiology as it is predominantly observed among older patients. One of the important MAPK signaling pathways was regulated by the reactive oxygen species (ROS) biological function induced by oxidative stressors [63]. The hub proteins response to chemical stress is an important part of many pathogenesis and neurodegenerative diseases and inflammation [64]. The drugs and medications used for anesthesia and surgery are involved in delirium under the chemical stress response. Among the MFs, most of the hub proteins are involved in kinase activities. In response to a wide range of stimuli, including mitogens, stress, heat shock, and proinflammatory cytokines, MAPKs activities control cellular responses [65, 66]. The MAPK signaling pathways might be crucial for delirium pathophysiology since the key delirium-associated hub proteins MAPK1, MAPK3, and MAPK14 were found to be associated with it. Besides that, the cellular senescence, lipid and atherosclerosis, shigellosis, developmental biology, various receptor associated pathways, cytokines and interleukins associated pathways and stress and inflammation related pathways were significantly shared by the delirium-associated hub-proteins. The shared signaling pathways consisted of the receptor-associated pathways reported by Takahaschi et al. 2020 [27]. ]. Furthermore, the current study found the stress and inflammation associated pathways shared by the hub-proteins which are also supported by the existing literature [4, 27, 67]. The GO analysis and the signaling pathway analysis revealed significant information about the molecular mechanism of delirium-associated hub-proteins that are essentially important for delirium-related molecular and pathophysiological knowledge.

The TFs and the miRNAs play significant roles in the protein translation from a specific gene. The hub-proteins are also regulated by the key transcriptional and post-transcriptional regulatory molecules that have been detected in this study. The TF FOXC1 is associated with neuroinflammation and neuronal apoptosis, whereas neuroinflammation is highly related to neurodegenerative complications such as Alzheimer’s disease, Dementia, and Parkinson’s disease [68–70]. Neuroglobin (NGB) gene expression is associated with neural disease (Alzheimer’s Disease) when the GATA2 TF works to regulate the NGB gene expression [71]. Studies suggest that the Yin Yang 1 (YY1) TF correlates with the central nervous system. The YY1 regulates a significant number of genes associated with the nervous system [72], hence it might have greater involvement with delirium pathophysiology. The Activator Protein 2 (AP-2) transcription factor (TF) family has a vital involvement in gene expression regulation and various cancers development with the other members in this family [73]. The SREBF1 TF is significantly associated with neuropsychiatric disorder schizophrenia [74]. On the other hand, among the post-transcriptional regulatory element miRNAs, the overexpression of miR-375 is associated with Alzheimer’s and Parkinson’s disease [75, 76] with the miR-17-5 and miR-17-5p being related to various cancer developments [3, 77, 78]. The miR-106a-5p miRNA, has a strong connection to the regulation of CD4 + T-cells and functions as a tumor suppressor, and miR-125b-5p is interrelated with suppressing PI3K/AKT pathway in bladder cancer [79] and the miR-125a-5p is connected with the macrophages inflammatory response [80].

The reported hub-proteins and their signaling pathways, TFs were also associated with the hallmarks of aging components since age is considered one of the most important and significant factors for delirium. Among the nine hallmarks of aging [40] the cellular senescence, loss of proteostasis, alteration in intercellular communication, genomic and epigenomic alteration are closely connected with the reported hub-protein’s functions and enriched signaling pathways. For example, the cellular senescence signaling pathway was significantly enriched by the eight hub-proteins. The cytokines and inflammatory related pathways, signal transduction, oxidative stress and telomerase activity are significant signaling pathways closely connected with the loss of proteostasis, alteration in intercellular communication hallmarks of aging.

The hub-proteins related computationally identified repurposable drugs were retrieved from the database. The predicted drug molecules including aspirin, irbesartan, ephedrine-(racemic), nedocromil, and guanidine were characterized as anti-inflammatory, stimulating the central nervous system, and neuroprotective medication as confirmed by the existing literature [81–86]. The drug molecules may play an important role for therapeutic development against delirium if they are investigated more extensively though clinical trials and various wet lab experiments. Our results therefore provide a platform for future investigations into the underlying mechanisms of delirium and therapeutic treatment development.

The overall discussion about the key hub-proteins and their regulatory molecules revealed that the reported biomarkers have an agglomerative and strong interconnection with the delirium-associated genes. The signaling pathway analysis highlighted the cytokines and inflammatory pathways, cellular senescence pathway, stress and telomerase activity pathways were significantly associated with the molecular pathophysiological mechanism of delirium. The reported drug target hub-proteins and their associated computationally identified repurposable drug molecules may open a new and extensive research dimension in the field of delirium drug development.

### Implications of this study

The implications of this study are far-reaching. Firstly, the results provide a comprehensive overview of the key drug target proteins associated with delirium, their associated regulatory molecules, and functional pathways. This allows for better understanding of the molecular mechanisms underlying delirium and for a better evaluation of drug candidates for the treatment of this disorder. Secondly, the meta-data analyses of existing evidence indicate the potential of new drug targets, which could be explored further for the development of novel therapeutics. Finally, the study provides a platform for future research, allowing for further investigations into the underlying mechanisms of delirium and the development of more effective treatments. If proven, the findings from this study could be used to inform clinical practice and public health policy concerning the prevention and management of delirium.

### Limitations of this study

Firstly, the study used a comprehensive seed proteomic dataset from a systematic literature review and CTD database. In both cases, the research team and the database have ensured the integrity of the genomic/proteomic information. Secondly, during the proteomic data collection, only association with delirium was considered and the magnitude of association (either positive or negative) was ignored in both sources. In this aspect, it is unknown whether the reported hub proteins are associated with delirium in their upregulated or downregulated condition. On the other hand, no rigorous study has been found that reported the comprehensive gene expression data or any transcriptomics dataset which could reveal up and downregulated genes/proteins associated with delirium. Moreover, the meta-data used in the study may not be comprehensive or up to date. The study may be limited by potential confounding factors that may influence the association between drug target proteins, regulatory molecules and functional pathways and the development of delirium.

## Conclusion

Since delirium is considered a multifactorial critical medical condition, its anatomic molecular functions and pathophysiology might be more diverse and complicated. The ongoing genomic research and studies revealed some snapshots of the disease’s molecular diversity. A wide range of transcriptomics data can be generated and analyzed to identify the important genes at the critical time point of delirious patients, revealing the differentially expressed genes and proteins. More studies should be undertaken to collect gene expression data and hence map out their functional pathways and molecular mechanisms rigorously. The current study carried out a multi-omics network-based system biology analysis using the comprehensive delirium associated genomic dataset. The study revealed the key hub-proteins on a highly interconnected PPI network. The signaling pathways analysis of the hub-proteins showed different significant pathways associated with delirium and the hallmarks of aging. The study also identified some important regulatory molecules linked to delirium associated hub-proteins. The biomolecules identified in this study may have potential association with delirium which will contribute to future research by enhancing our understanding of the delirium mechanism. Moreover, the computationally predicted repurposable drug molecules associated with the hub-proteins as reported in this study provide a wide range of research opportunities in therapeutic developments against delirium. The study will also provide a platform for future investigations into the underlying mechanisms of delirium.

### Electronic supplementary material

Below is the link to the electronic supplementary material.

Supplementary Material 1

Supplementary Material 2

Supplementary Material 3

## Data Availability

All data generated or analyzed during this study are either publicly available or included in this article.
