# Supplementary material for: Exploration of key drug target proteins highlighting their related regulatory molecules, functional pathways and drug candidates associated with delirium: evidence from meta-data analyses
Source: BMC Geriatr. 2023 Nov 22;23:767. doi: 10.1186/s12877-023-04457-1 (PMC10666371; doi:10.1186/s12877-023-04457-1)
Supplement: Supplementary file 2 — Supplementary Material 2 [file 12877_2023_4457_MOESM2_ESM.docx]

**Systematic literature review for delirium-associated proteomic data collection**

***Rationale and Objectives***

Delirium is a serious neuropsychiatric medical condition triggered by multiple predisposing and precipitating factors, including critical medical situations, drug usage or withdrawal, and major surgery. The delirium-associated pathophysiological pathways and the key driver biomarkers are still in the dark. Due to the lack of gene expression data of delirium, no rigorous gene expression data or transcriptomics data were available to identify the key genomic biomarkers. Although, demand-based gene expression analysis was conducted for few targeted genes and reported the associated genes of delirium. To make a comprehensive genomic dataset of delirium we have introduced a systematic review to collect the gene expression dataset of delirium collected from distinct small gene expression studies.

***Search strategy***

We conducted a systematic review of the literature to identify research on delirium-associated proteomic biomarkers that were published between January 1, 2000, and December 31, 2022, using the electronic bibliographic databases PubMed, Scopus, and EBSCOhost (CINAHL, Medline). A total of 2065 studies were identified and reviewed, and 54 studies were included in our study (Figure 1).

A comprehensive electronic literature search was conducted on the selected databases using the MeSH terms, keywords, and subject headings. Only studies that were published in the journal between 1 January 2000 and 31 December 2022 were considered for screening. The primary keywords were “delirium” and “biomarker” used along with a combination of other associated keywords including “marker”, ‘genetic”, “proteomic”, “genes” and “protein” to search the studies. Boolean operators “AND”, and “OR” were applied to
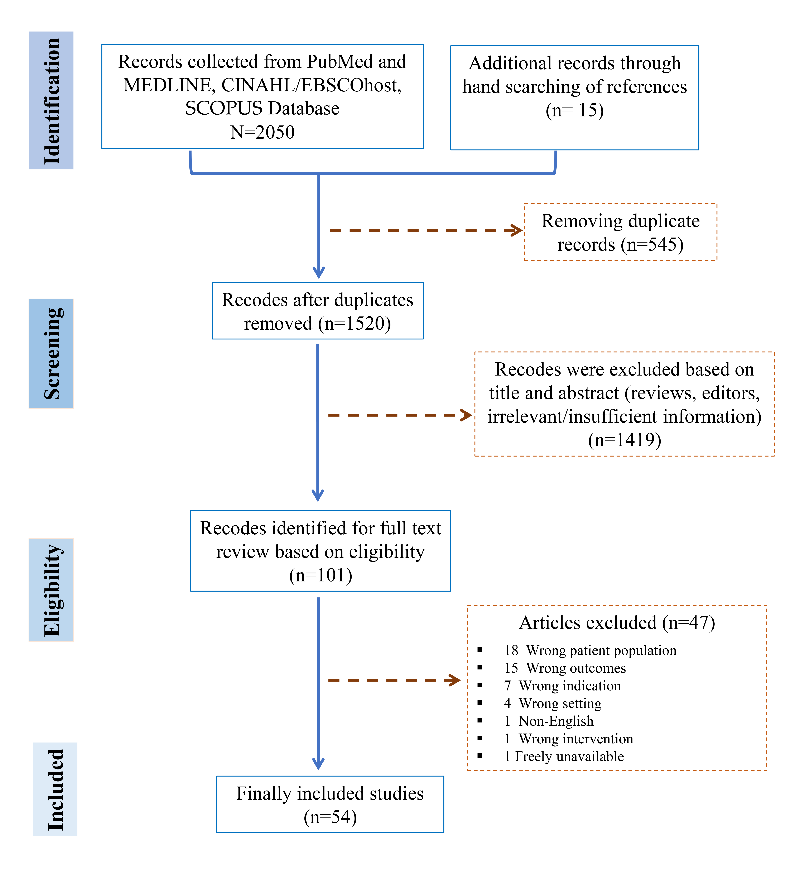
combine the searching keywords. In addition, this review was complemented by a thorough manual search of related studies. Further, studies were identified through citation searches of included studies and manual searches for professional web sources and key journals in these fields of research.

Figure 1: The PRISMA Flowchart of the current study.

***Eligibility/Selection criteria***

Eligible studies were included if they were i) based on original research studies focused and reported on genes/proteins showing any relationship between delirium and genes/proteins; ii) assessed and validated using established methods; and iii) were published between January 1, 2000, and December 31, 2022, in English. Otherwise, editorials, letters, perspectives, commentaries, reports, reviews, study protocols, publications in other languages, and studies with 'insufficient related data' were excluded.

***Study screening and selection***

The eligibility of studies to be included was determined following a three-stage screening process. The first stage involved screening of studies by title to eliminate duplications. The second stage required reading abstracts to determine their relevance to our study. Finally, the third stage necessitated reading full texts of the retained studies, and those that met the set criteria were kept. The first author, MPM carried out and recorded the above process and shared the record with KA, JW and RAM for verification. Discrepancies were discussed and resolved by consensus. On the data collection stage, MPM were collected the delirium-associated genomic data and shared with other researcher for review. If any confusion came, we discussed in group to take the final decision.

***Quality assessment***

The quality assessment of the included studies was conducted because of the heterogeneity among the study designs of the included studies. In this systematic review, cohort, case-control, cross-sectional, randomized control trials, and longitudinal study designs were found among the included studies. The Joanna Briggs Institute (JBI) [1] provided critical quality assessment tools that have been utilized in this study for quality assessment. The JBI quality appraisal tools are widely used in academic studies to assess the risk of bias (graded as high, moderate, or low) [2–5] where the higher quality scores demonstrate better confidence and vice versa. The first JBI appraisal tool was used to evaluate the 38 cohort studies included in this review. The checklist evaluates key study methodology components for bias in the study conception, execution, and analytics parts. The other consecutive JBI checklists were applied to evaluate ten case controls, three randomized control trials, two cross-sectional, and a longitudinal study included in our review. Among the included studies, most of the studies were of medium/moderate (n=37/54) and high quality (n=17/54) based on appraisal score, indicating the robustness of the included studies.

***Data collection***

The delirium associated genes were collected from the selected articles without their mode of association (positive/negative association). The data extraction summary has been provided in the following table.

**Result Table: The basic summary information table of the selected studies.**

| Authors & year | Country | Type of Study | Delirium Assessment Methods | All Associated Proteins/Genes |
| --- | --- | --- | --- | --- |
| Adamis et al, 2009 [6] | Ireland | Longitudinal Study | CAM | IGF-1, IL-1ra, IFN-γ |
| Egberts et al, 2015 [7] | Netherlands | Case-Control Study | DSM-IV | Neopterin, IL-6, IGF-1 |
| Hirsch et al, 2016 [8] | USA | Cohort Study | CAM | IL-6, RAGE, IL-5, IL-8, MCP-1, IL-10, IFN-a, IL-4, IGN-gamma, IL-12 |
| Kazmierski J et al, 2014 [9] | Poland | Cohort Study | CAM-ICU | IL-2, TNF-a |
| Miao et al, 2018 [10] | China | Cohort Study | DSM‑IV | Neopterin, CRP, IL-6, IGF-1 |
| Ritter et al, 2014 [11] | Brazil | Cohort Study | CAM-ICU | STNFR1, STNFR2, adiponectin, IL-1b |
| Sun et al, 2016 [12] | China | Cohort Study | CAM | IL-6, CRP, procalcitonin, Cortisol |
| Van Munster et al, 2008 [13] | Netherlands | Cohort Study | CAM | IL-6, IL-8 |
| Vasunilashorn SM et al, 2015 [14] | USA | Case-Control Study | CAM | IL-6, I-2, TNF-a |
| Westhoff et al, 2013 [15] | Netherlands | Cohort Study | CAM | IL-6, IL-ra, FLT-31 |
| Heinrich M et al, 2021 [16] | Germany and Netherlands | Cohort Study | CAM | CHRM2, CHRM4 |
| Van Munster et al, 2010 [17] | Netherlands | Cohort Study | CAM | DRD2, DRD3, SLC6A3 |
| Terrelonge M et al, 2022 [18] | USA | Case-Control Study | CAM | FKBP5, KIBRA, KLOTHO, MTNR1B, SIRT1 |
| Yamanashi T et al, 2021 [19] | USA | Cohort Study | CAM-ICU | TNF |
| Yamanashi T et al, 2021 [20] | USA | Cohort Study | CAM-ICU | TNF-a, IL-1b, IL-6 |
| Steimer M et al, 2021 [21] | Germany | Cohort Study | nuDesc | PER2, HO1 |
| Nekrosius D et al, 2019 [22] | Lithuania | Cohort Study | CAM | COMT |
| Rhee J et al, 2021 [23] | USA | Randomized Control Trial | CAM | FN1.4, FN1.3, Troponin 1, C5a, IL-1, Cadherin-12, IL-6, PKC-Z, FGF-16, TIMP-1 |
| Ballweg T et al, 2021 [24] | USA | Cohort Study | CAM-ICU | IL-8, IL-10 |
| Tang C et al, 2020 [25] | China | Randomized Control Trial | CAM | IL-6, TNF-a, IL-10 |
| Vasunilashorn SM et al, 2019 [26] | USA | Case-Control Study | CAM | IL-6, IL-2, CRP, SERPINA3, HPX, ORM1, AZGP1 |
| Van Munster BC et al, 2010 [27] | Netherlands | Cohort Study | CAM | Cortisol, IL-6, IL-8, S100B |
| Peters van Ton AM et al, 2020 [28] | Netherlands | Case-Control Study | DSM-IV | NBL1, THY1, NrCAM, NCAN, TNKRSF21, DINER, CADM3, RGMA, CTSS, IL-1a, RSPO1, N-Cdase, WFIKKN1, HAGH, CD200R1, IL-5ra, ENRAGE, MCP4, CRTAM, NEP, CST5, BCAN, CASP8, EFNA4, SCF, EZR, CX3CL1, HGF, TGF-B1, SMOC2, CXCL6 |
| Vasunilashorn SM et al, 2022 [29] | USA | Case-Control Study | CAM | CHI3L1, PF4, MICA, ADCYAP1, RETN, CD300C, CD274, FCGR3B, PAPPA, TNFRSF1A, PLA2G2A, IL-6, TIMP1, THBS1, CD177, CKM, NAAA, ANP32B, KLKB1, BMP1, C4A, CCL27, CTSV, TNFSF9, CCL11, IL-25, AMN, STX1A, CCL16, CHKB, SERPING1, MIA, CCL27, CDH1, PLG, LRIG3 |
| Ka´zmierski J et al, 2021 [30] | Poland | Cohort Study | CAM | MCP-1, CRP |
| Ye C et al, 2020 [31] | China | Cohort Study | CAM-ICU | IL-6, CHI3L1, S100B, Lp-PLA2, MIF, ICAM-1, VCAM-1, BACE1, a-SYN |
| Ritchie CW et al, 2014 [32] | UK | Cross-Sectional Study | CAM | CRP |
| Plaschke K et al, 2010 [33] | Germany | Cohort Study | CAM-ICU | IL-6, Cortisol |
| Szwed K et al, 2021 [34] | Poland | Case-Control Study | CAM-ICU | NSP, GFAP |
| Erikson K et al, 2019 [35] | Finland | Cohort Study | CAM‐ICU | S100B, IL-6 |
| Yuan Y et al, 2020 [36] | China | Case-Control Study | CAM | IL-1b, IL-6, a-SYN |
| Khan SH et al, 2022 [37] | USA | Randomized Control Trial | CAM‐ICU | CRP, IL-8, IL-10 |
| Menzenbach J et al, 2021 [38] | Germany | Cohort Study | CAM‐ICU | IL-8, CCL2, RAGE, Resistin, ANGPT2, TIE2, THBD, SDC-1, E-Sel, VCAM-1, ICAM-1, CXCL5, NSE, RAGE |
| Boogaard MVD et al, 2011 [39] | Netherlands | Cross-Sectional Study | CAM-ICU | IL-8, MCP-1, PCT, Cortisol, and S100B, IL-8, IL-1, IL-10 |
| Hall RJ et al, 2013 [40] | Scotland | Cohort Study | CAM | S100B |
| Xu WB et al, 2019 [41] | China | Case-Control Study | CAM | sFGL2 |
| Chai Lv et al, 2021 [42] | China | Cohort Study | CAM | IL-6 |
| Khan BA et al, 2013 [43] | USA | Cohort Study | CAM-ICU | S100B |
| Mao M et al, 2022 [44] | China | Cohort Study | CAM | PGE2, NfL, S100B, GFAP |
| Khan BA et al, 2020 [45] | USA | Cohort Study | CAM-ICU | IL-6, IL-8, IL-10, TNF-a, CRP, S100B |
| Neerland et al, 2016 [46] | Norway | Cohort Study | CAM | CRP, sIL-6R |
| Girard TD et al, 2012 [47] | USA | Cohort Study | CAM-ICU | MMP-9, Protein C, sTNFR1 |
| Maes M et al, 2022 [48] | Thailand | Cohort Study | DRSR-98 | GlutaR, AQP4, HSP60 |
| Pfister D et al, 2008 [49] | USA | Cohort Study | CAM-ICU | CRP, S100B, Cortisol |
| Cerejeira J et al, 2012 [50] | Portugal | Cohort Study | CAM | CRP, IL-6, IL-8, IL-10 |
| Rooij SE et al, 2007 [51] | Netherlands | Cohort Study | CAM | IL-6, IL-8 |
| Wang B et al, 2022 [52] | China | Case-Control Study | CAM | IGF-1, APP |
| McNeil JB et al, 2019 [53] | USA | Cohort Study | CAM-ICU | PAI-1, IL-6 |
| Klimiec Moskal et al, 2021 [54] | Poland | Cohort Study | CAM-ICU | Gal-3BP |
| Cape E et al, 2014 [55] | Netherlands | Cohort Study | CAM | IL-1b, IL-1ra, MCP-1 |
| Lindblom RPF et al, 2018 [56] | Sweden | Cohort Study | CAM-ICU | TR4, EZH2, CHI3L1, IL-6, SFRP2, PMP2, RTN4R, GFAP, CX3CL1, ICAM-1 |
| Skrede et al, 2015 [57] | Norway | Cohort Study | CAM | MCP-1 |
| Brattinga B et al, 2022 [58] | Netherlands | Cohort Study | DOS scale | IL-10, NGAL |
| Chen Y et al, 2019 [59] | China | Cohort Study | CAM-ICU | IL-6 |

References

1. Joanna Briggs Institute (JBI) JBI’s Critical Appraisal Tools. *Fac. Heal. Med. Sci. Univ. Adelaide SA 5006 Adelaide, Aust.* **2022**, 2–6.

2. Mahumud, R.A.; Kamara, J.K.; Renzaho, A.M.N. The Epidemiological Burden and Overall Distribution of Chronic Comorbidities in Coronavirus Disease-2019 among 202,005 Infected Patients: Evidence from a Systematic Review and Meta-Analysis. *Infection* 2020, *48*, 813–833.

3. Porto De Toledo, I.; Stefani, F.M.; Porporatti, A.L.; Mezzomo, L.A.; Peres, M.A.; Flores-Mir, C.; De Luca Canto, G. Prevalence of Otologic Signs and Symptoms in Adult Patients with Temporomandibular Disorders: A Systematic Review and Meta-Analysis. *Clin. Oral Investig.* **2017**, *21*, 597–605, doi:10.1007/s00784-016-1926-9.

4. Munn, Z.; MClinSc, S.M.; Lisy, K.; Riitano, D.; Tufanaru, C. Methodological Guidance for Systematic Reviews of Observational Epidemiological Studies Reporting Prevalence and Cumulative Incidence Data. *Int. J. Evid. Based. Healthc.* **2015**, *13*, 147–153, doi:10.1097/XEB.0000000000000054.

5. Mahumud, R.A.; Ali, M.A.; Kundu, S.; Rahman, M.A.; Kamara, J.K.; Renzaho, A.M.N. Effectiveness of COVID-19 Vaccines against Delta Variant (B.1.617.2): A Meta-Analysis. *Vaccines* **2022**, *10*, 277, doi:10.3390/vaccines10020277.

6. Adamis, D.; Lunn, M.; Martin, F.C.; Treloar, A.; Gregson, N.; Hamilton, G.; Macdonald, A.J.D. Cytokines and IGF-I in Delirious and Non-Delirious Acutely Ill Older Medical Inpatients. *Age Ageing* **2009**, *38*, 326–332; discussion 251, doi:10.1093/ageing/afp014.

7. Egberts, A.; Wijnbeld, E.H.A.; Fekkes, D.; van der Ploeg, M.A.; Ziere, G.; Hooijkaas, H.; van der Cammen, T.J.M.; Mattace-Raso, F.U.S. Neopterin: A Potential Biomarker for Delirium in Elderly Patients. *Dement. Geriatr. Cogn. Disord.* **2015**, *39*, 116–124, doi:10.1159/000366410.

8. Hirsch, J.; Vacas, S.; Terrando, N.; Yuan, M.; Sands, L.P.; Kramer, J.; Bozic, K.; Maze, M.M.; Leung, J.M. Perioperative Cerebrospinal Fluid and Plasma Inflammatory Markers after Orthopedic Surgery. *J. Neuroinflammation* **2016**, *13*, 211, doi:10.1186/s12974-016-0681-9.

9. Kazmierski, J.; Banys, A.; Latek, J.; Bourke, J.; Jaszewski, R. Raised IL-2 and TNF-α Concentrations Are Associated with Postoperative Delirium in Patients Undergoing Coronary-Artery Bypass Graft Surgery. *Int. Psychogeriatrics* **2014**, *26*, 845–855, doi:10.1017/S1041610213002378.

10. Miao, S.; Shen, P.; Zhang, Q.; Wang, H.; Shen, J.; Wang, G.; Lv, D. Neopterin and Mini-Mental State Examination Scores, Two Independent Risk Factors for Postoperative Delirium in Elderly Patients with Open Abdominal Surgery. *J. Cancer Res. Ther.* **2018**, *14*, 1234–1238, doi:10.4103/0973-1482.192764.

11. Ritter, C.; Tomasi, C.D.; Dal-Pizzol, F.; Pinto, B.B.; Dyson, A.; de Miranda, A.S.; Comim, C.M.; Soares, M.; Teixeira, A.L.; Quevedo, J.; et al. Inflammation Biomarkers and Delirium in Critically Ill Patients. *Crit. Care* **2014**, *18*, R106, doi:10.1186/cc13887.

12. Sun, L.; Jia, P.; Zhang, J.; Zhang, X.; Zhang, Y.; Jiang, H.; Jiang, W.; Guo, Y. Production of Inflammatory Cytokines, Cortisol, and A&beta;1-40 in Elderly Oral Cancer Patients with Postoperative Delirium. *Neuropsychiatr. Dis. Treat.* **2016**, *Volume 12*, 2789–2795, doi:10.2147/NDT.S113077.

13. Van Munster, B.C.; Korevaar, J.C.; Zwinderman, A.H.; Levi, M.; Wiersinga, W.J.; De Rooij, S.E. Time-Course of Cytokines during Delirium in Elderly Patients with Hip Fractures. *J. Am. Geriatr. Soc.* **2008**, *56*, 1704–1709, doi:10.1111/j.1532-5415.2008.01851.x.

14. Vasunilashorn, S.M.; Ngo, L.; Inouye, S.K.; Libermann, T.A.; Jones, R.N.; Alsop, D.C.; Guess, J.; Jastrzebski, S.; McElhaney, J.E.; Kuchel, G.A.; et al. Cytokines and Postoperative Delirium in Older Patients Undergoing Major Elective Surgery. *Journals Gerontol. - Ser. A Biol. Sci. Med. Sci.* **2014**, *70*, 1289–1295, doi:10.1093/gerona/glv083.

15. Westhoff, D.; Witlox, J.; Koenderman, L.; Kalisvaart, K.J.; de Jonghe, J.F.M.; van Stijn, M.F.M.; Houdijk, A.P.J.; Hoogland, I.C.M.; MacLullich, A.M.J.; van Westerloo, D.J.; et al. Preoperative Cerebrospinal Fluid Cytokine Levels and the Risk of Postoperative Delirium in Elderly Hip Fracture Patients. *J. Neuroinflammation* **2013**, *10*, 122, doi:10.1186/1742-2094-10-122.

16. Heinrich, M.; Sieg, M.; Kruppa, J.; Nürnberg, P.; Schreier, P.H.; Heilmann-Heimbach, S.; Hoffmann, P.; Nöthen, M.M.; Janke, J.; Pischon, T.; et al. Association between Genetic Variants of the Cholinergic System and Postoperative Delirium and Cognitive Dysfunction in Elderly Patients. *BMC Med. Genomics* **2021**, *14*, 248, doi:10.1186/s12920-021-01071-1.

17. van Munster, B.C.; Yazdanpanah, M.; Tanck, M.W.T.; de Rooij, S.E.J.A.; van de Giessen, E.; Sijbrands, E.J.G.; Zwinderman, A.H.; Korevaar, J.C. Genetic Polymorphisms in the DRD2, DRD3, and SLC6A3 Gene in Elderly Patients with Delirium. *Am. J. Med. Genet. B. Neuropsychiatr. Genet.* **2010**, *153B*, 38–45, doi:10.1002/ajmg.b.30943.

18. Terrelonge, M.; LaHue, S.C.; Tang, C.; Movsesyan, I.; Pullinger, C.R.; Dubal, D.B.; Leung, J.; Douglas, V.C. KIBRA, MTNR1B, and FKBP5 Genotypes Are Associated with Decreased Odds of Incident Delirium in Elderly Post-Surgical Patients. *Sci. Rep.* **2022**, *12*, 556, doi:10.1038/s41598-021-04416-z.

19. Yamanashi, T.; Saito, T.; Yu, T.; Alario, A.; Comp, K.; Crutchley, K.J.; Sullivan, E.J.; Anderson, Z.-E.M.; Marra, P.S.; Chang, G.; et al. DNA Methylation in the TNF-Alpha Gene Decreases along with Aging among Delirium Inpatients. *Neurobiol. Aging* **2021**, *105*, 310–317, doi:10.1016/j.neurobiolaging.2021.05.005.

20. Yamanashi, T.; Nagao, T.; Wahba, N.E.; Marra, P.S.; Crutchley, K.J.; Meyer, A.A.; Andreasen, A.J.; Hellman, M.M.; Jellison, S.S.; Hughes, C.G.; et al. DNA Methylation in the Inflammatory Genes after Neurosurgery and Diagnostic Ability of Post-Operative Delirium. *Transl. Psychiatry* **2021**, *11*, 627, doi:10.1038/s41398-021-01752-6.

21. Steimer, M.; Kaiser, S.; Ulbrich, F.; Kalbhenn, J.; Bürkle, H.; Schallner, N. Expression of HO1 and PER2 Can Predict the Incidence of Delirium in Trauma Patients with Concomitant Brain Injury. *Sci. Rep.* **2021**, *11*, 15388, doi:10.1038/s41598-021-94773-6.

22. Nekrosius, D.; Kaminskaite, M.; Jokubka, R.; Pranckeviciene, A.; Lideikis, K.; Tamasauskas, A.; Bunevicius, A. Association of COMT Val158Met Polymorphism With Delirium Risk and Outcomes After Traumatic Brain Injury. *J. Neuropsychiatry Clin. Neurosci.* **2019**, *31*, 298–305, doi:10.1176/appi.neuropsych.18080195.

23. Rhee, J.; Kuznetsov, A.; McKay, T.; Lyons, M.; Houstis, N.; Mekkonen, J.; Ethridge, B.; Ibala, R.; Hahm, E.; Gitlin, J.; et al. Serum Proteomics of Older Patients Undergoing Major Cardiac Surgery: Identification of Biomarkers Associated With Postoperative Delirium. *Front. Aging Neurosci.* **2021**, *13*, 1–11, doi:10.3389/fnagi.2021.699763.

24. Ballweg, T.; White, M.; Parker, M.; Casey, C.; Bo, A.; Farahbakhsh, Z.; Kayser, A.; Blair, A.; Lindroth, H.; Pearce, R.A.; et al. Association between Plasma Tau and Postoperative Delirium Incidence and Severity: A Prospective Observational Study. *Br. J. Anaesth.* **2021**, *126*, 458–466, doi:10.1016/j.bja.2020.08.061.

25. Tang, C.; Hu, Y.; Zhang, Z.; Wei, Z.; Wang, H.; Geng, Q.; Shi, S.; Wang, S.; Wang, J.; Chai, X. Dexmedetomidine with Sufentanil in Intravenous Patient-Controlled Analgesia for Relief from Postoperative Pain, Inflammation and Delirium after Esophageal Cancer Surgery. *Biosci. Rep.* **2020**, *40*, 1–12, doi:10.1042/BSR20193410.

26. Vasunilashorn, S.M.; Ngo, L.H.; Chan, N.Y.; Zhou, W.; Dillon, S.T.; Otu, H.H.; Inouye, S.K.; Wyrobnik, I.; Kuchel, G.A.; McElhaney, J.E.; et al. Development of a Dynamic Multi-Protein Signature of Postoperative Delirium. *J. Gerontol. A. Biol. Sci. Med. Sci.* **2019**, *74*, 261–268, doi:10.1093/gerona/gly036.

27. van Munster, B.C.; Bisschop, P.H.; Zwinderman, A.H.; Korevaar, J.C.; Endert, E.; Wiersinga, W.J.; van Oosten, H.E.; Goslings, J.C.; de Rooij, S.E.J.A. Cortisol, Interleukins and S100B in Delirium in the Elderly. *Brain Cogn.* **2010**, *74*, 18–23, doi:10.1016/j.bandc.2010.05.010.

28. Peters van Ton, A.M.; Verbeek, M.M.; Alkema, W.; Pickkers, P.; Abdo, W.F. Downregulation of Synapse-Associated Protein Expression and Loss of Homeostatic Microglial Control in Cerebrospinal Fluid of Infectious Patients with Delirium and Patients with Alzheimer’s Disease. *Brain. Behav. Immun.* **2020**, *89*, 656–667, doi:10.1016/j.bbi.2020.06.027.

29. Vasunilashorn, S.M.; Dillon, S.T.; Chan, N.Y.; Fong, T.G.; Joseph, M.; Tripp, B.; Xie, Z.; Ngo, L.H.; Lee, C.G.; Elias, J.A.; et al. Proteome-Wide Analysis Using SOMAscan Identifies and Validates Chitinase-3-Like Protein 1 as a Risk and Disease Marker of Delirium Among Older Adults Undergoing Major Elective Surgery. *J. Gerontol. A. Biol. Sci. Med. Sci.* **2022**, *77*, 484–493, doi:10.1093/gerona/glaa326.

30. Kaźmierski, J.; Miler, P.; Pawlak, A.; Jerczyńska, H.; Woźniak, J.; Frankowska, E.; Brzezińska, A.; Woźniak, K.; Krejca, M.; Wilczyński, M. Elevated Monocyte Chemoattractant Protein-1 as the Independent Risk Factor of Delirium after Cardiac Surgery. A Prospective Cohort Study. *J. Clin. Med.* **2021**, *10*, 1587, doi:10.3390/jcm10081587.

31. Ye, C.; Zhang, Y.; Luo, S.; Cao, Y.; Gao, F.; Wang, E. Correlation of Serum BACE1 With Emergence Delirium in Postoperative Patients: A Preliminary Study. *Front. Aging Neurosci.* **2020**, *12*, 1–7, doi:10.3389/fnagi.2020.555594.

32. Ritchie, C.W.; Newman, T.H.; Leurent, B.; Sampson, E.L. The Association between C-Reactive Protein and Delirium in 710 Acute Elderly Hospital Admissions. *Int. psychogeriatrics* **2014**, *26*, 717–724, doi:10.1017/S1041610213002433.

33. Plaschke, K.; Fichtenkamm, P.; Schramm, C.; Hauth, S.; Martin, E.; Verch, M.; Karck, M.; Kopitz, J. Early Postoperative Delirium after Open-Heart Cardiac Surgery Is Associated with Decreased Bispectral EEG and Increased Cortisol and Interleukin-6. *Intensive Care Med.* **2010**, *36*, 2081–2089, doi:10.1007/s00134-010-2004-4.

34. Szwed, K.; Szwed, M.; Kozakiewicz, M.; Karłowska-Pik, J.; Soja-Kukieła, N.; Bartoszewska, A.; Borkowska, A. Circulating MicroRNAs and Novel Proteins as Potential Biomarkers of Neurological Complications after Heart Bypass Surgery. *J. Clin. Med.* **2021**, *10*, 3091, doi:10.3390/jcm10143091.

35. Erikson, K.; Ala-Kokko, T.I.; Koskenkari, J.; Liisanantti, J.H.; Kamakura, R.; Herzig, K.H.; Syrjälä, H. Elevated Serum S-100β in Patients with Septic Shock Is Associated with Delirium. *Acta Anaesthesiol. Scand.* **2019**, *63*, 69–73, doi:10.1111/aas.13228.

36. Yuan, Y.; Li, Z.; Yang, N.; Han, Y.; Ji, X.; Han, D.; Wang, X.; Li, Y.; Liu, T.; Yuan, F.; et al. Exosome α-Synuclein Release in Plasma May Be Associated With Postoperative Delirium in Hip Fracture Patients. *Front. Aging Neurosci.* **2020**, *12*, 1–10, doi:10.3389/fnagi.2020.00067.

37. Khan, S.H.; Lindroth, H.; Jawed, Y.; Wang, S.; Nasser, J.; Seyffert, S.; Naqvi, K.; Perkins, A.J.; Gao, S.; Kesler, K.; et al. Serum Biomarkers in Postoperative Delirium After Esophagectomy. *Ann. Thorac. Surg.* **2022**, *113*, 1000–1007, doi:10.1016/j.athoracsur.2021.03.035.

38. Menzenbach, J.; Frede, S.; Petras, J.; Guttenthaler, V.; Kirfel, A.; Neumann, C.; Mayr, A.; Wittmann, M.; Coburn, M.; Klaschik, S.; et al. Perioperative Vascular Biomarker Profiling in Elective Surgery Patients Developing Postoperative Delirium: A Prospective Cohort Study. *Biomedicines* **2021**, *9*, 553, doi:10.3390/biomedicines9050553.

39. van den Boogaard, M.; Kox, M.; Quinn, K.L.; van Achterberg, T.; van der Hoeven, J.G.; Schoonhoven, L.; Pickkers, P. Biomarkers Associated with Delirium in Critically Ill Patients and Their Relation with Long-Term Subjective Cognitive Dysfunction; Indications for Different Pathways Governing Delirium in Inflamed and Noninflamed Patients. *Crit. Care* **2011**, *15*, R297, doi:10.1186/cc10598.

40. Hall, R.J.; Ferguson, K.J.; Andrews, M.; Green, A.J.E.; White, T.O.; Armstrong, I.R.; Maclullich, A.M.J. Delirium and Cerebrospinal Fluid S100B in Hip Fracture Patients: A Preliminary Study. *Am. J. Geriatr. Psychiatry* **2013**, *21*, 1239–1243, doi:10.1016/j.jagp.2012.12.024.

41. Xu, W. Bin; Hu, Q.H.; Wu, C.N.; Fan, Z.K.; Song, Z.F. Serum Soluble Fibrinogen-like Protein 2 Concentration Predicts Delirium after Acute Pancreatitis. *Brain Behav.* **2019**, *9*, e01261, doi:10.1002/brb3.1261.

42. Lv, X.C.; Lin, Y.; Wu, Q. song; Wang, L.; Hou, Y. ting; Dong, Y.; Chen, L. wan Plasma Interleukin-6 Is a Potential Predictive Biomarker for Postoperative Delirium among Acute Type a Aortic Dissection Patients Treated with Open Surgical Repair. *J. Cardiothorac. Surg.* **2021**, *16*, 146, doi:10.1186/s13019-021-01529-4.

43. Khan, B.A.; Farber, M.O.; Campbell, N.; Perkins, A.; Prasad, N.K.; Hui, S.L.; Miller, D.K.; Calvo-Ayala, E.; Buckley, J.D.; Ionescu, R.; et al. S100 Calcium Binding Protein B as a Biomarker of Delirium Duration in the Intensive Care Unit - An Exploratory Analysis. *Int. J. Gen. Med.* **2013**, *6*, 855–861, doi:10.2147/IJGM.S51004.

44. Mao, M.; Wang, L. yuan; Zhu, L. yue; Wang, F.; Ding, Y.; Tong, J. hua; Sun, J.; Sun, Q.; Ji, M. huo Higher Serum PGE2 Is a Predicative Biomarker for Postoperative Delirium Following Elective Orthopedic Surgery in Elderly Patients. *BMC Geriatr.* **2022**, *22*, 685, doi:10.1186/s12877-022-03367-y.

45. Khan, B.A.; Perkins, A.J.; Prasad, N.K.; Shekhar, A.; Campbell, N.L.; Gao, S.; Wang, S.; Khan, S.H.; Marcantonio, E.R.; Twigg, H.L.; et al. Biomarkers of Delirium Duration and Delirium Severity in the ICU. *Crit. Care Med.* **2020**, *48*, 353–361, doi:10.1097/CCM.0000000000004139.

46. Neerland, B.E.; Hall, R.J.; Seljeflot, I.; Frihagen, F.; MacLullich, A.M.J.; Raeder, J.; Wyller, T.B.; Watne, L.O. Associations Between Delirium and Preoperative Cerebrospinal Fluid C-Reactive Protein, Interleukin-6, and Interleukin-6 Receptor in Individuals with Acute Hip Fracture. *J. Am. Geriatr. Soc.* **2016**, *64*, 1456–1463, doi:10.1111/jgs.14238.

47. Girard, T.D.; Ware, L.B.; Bernard, G.R.; Pandharipande, P.P.; Thompson, J.L.; Shintani, A.K.; Jackson, J.C.; Dittus, R.S.; Ely, E.W. Associations of Markers of Inflammation and Coagulation with Delirium during Critical Illness. *Intensive Care Med.* **2012**, *38*, 1965–1973, doi:10.1007/s00134-012-2678-x.

48. Maes, M.; Thisayakorn, P.; Thipakorn, Y.; Tantavisut, S.; Sirivichayakul, S.; Vojdani, A. Reactivity to Neural Tissue Epitopes, Aquaporin 4 and Heat Shock Protein 60 Is Associated with Activated Immune–Inflammatory Pathways and the Onset of Delirium Following Hip Fracture Surgery. *Eur. Geriatr. Med.* **2022**, *14*, 99–112, doi:10.1007/s41999-022-00729-y.

49. Pfister, D.; Siegemund, M.; Dell-Kuster, S.; Smielewski, P.; Rüegg, S.; Strebel, S.P.; Marsch, S.C.U.; Pargger, H.; Steiner, L.A. Cerebral Perfusion in Sepsis-Associated Delirium. *Crit. Care* **2008**, *12*, R63, doi:10.1186/cc6891.

50. Cerejeira, J.M.S.; Nogueira, V.; Luís, P.; Vaz-Serra, A.; Mukaetova-Ladinska, E.B. The Cholinergic System and Inflammation: Common Pathways in Delirium Pathophysiology. *J. Am. Geriatr. Soc.* **2012**, *60*, 669–675, doi:10.1111/j.1532-5415.2011.03883.x.

51. de Rooij, S.E.; van Munster, B.C.; Korevaar, J.C.; Levi, M. Cytokines and Acute Phase Response in Delirium. *J. Psychosom. Res.* **2007**, *62*, 521–525, doi:10.1016/j.jpsychores.2006.11.013.

52. Wang, B.; Yin, Z.; Lin, Y.; Deng, X.; Liu, F.; Tao, H.; Dong, R.; Lin, X.; Bi, Y. Correlation between MicroRNA-320 and Postoperative Delirium in Patients Undergoing Tibial Fracture Internal Fixation Surgery. *BMC Anesthesiol.* **2022**, *22*, 1–12, doi:10.1186/s12871-022-01612-w.

53. McNeil, J.B.; Hughes, C.G.; Girard, T.; Ware, L.B.; Ely, E.W.; Chandrasekhar, R.; Han, J.H. Plasma Biomarkers of Inflammation, Coagulation, and Brain Injury as Predictors of Delirium Duration in Older Hospitalized Patients. *PLoS One* **2019**, *14*, e0226412, doi:10.1371/journal.pone.0226412.

54. Klimiec-Moskal, E.; Pasinska, P.; Kowalska, K.; Klimkowicz-Mrowiec, A.; Pera, J.; Slowik, A.; Dziedzic, T. Elevated Plasma Levels of Galectin-3 Binding Protein Are Associated with Post-Stroke Delirium - A Pilot Study. *J. Neuroimmunol.* **2021**, *356*, 577579, doi:10.1016/j.jneuroim.2021.577579.

55. Cape, E.; Hall, R.J.; van Munster, B.C.; de Vries, A.; Howie, S.E.M.; Pearson, A.; Middleton, S.D.; Gillies, F.; Armstrong, I.R.; White, T.O.; et al. Cerebrospinal Fluid Markers of Neuroinflammation in Delirium: A Role for Interleukin-1β in Delirium after Hip Fracture. *J. Psychosom. Res.* **2014**, *77*, 219–225, doi:10.1016/j.jpsychores.2014.06.014.

56. Lindblom, R.P.F.; Shen, Q.; Axén, S.; Landegren, U.; Kamali-Moghaddam, M.; Thelin, S. Protein Profiling in Serum and Cerebrospinal Fluid Following Complex Surgery on the Thoracic Aorta Identifies Biological Markers of Neurologic Injury. *J. Cardiovasc. Transl. Res.* **2018**, *11*, 503–516, doi:10.1007/s12265-018-9835-8.

57. Skrede, K.; Wyller, T.B.; Watne, L.O.; Seljeflot, I.; Juliebø, V. Is There a Role for Monocyte Chemoattractant Protein-1 in Delirium? Novel Observations in Elderly Hip Fracture Patients. *BMC Res. Notes* **2015**, *8*, 1–4, doi:10.1186/s13104-015-1129-5.

58. Brattinga, B.; Plas, M.; Spikman, J.M.; Rutgers, A.; De Haan, J.J.; Absalom, A.R.; Van Der Wal-Huisman, H.; De Bock, G.H.; Van Leeuwen, B.L. The Association between the Inflammatory Response Following Surgery and Post-Operative Delirium in Older Oncological Patients: A Prospective Cohort Study. *Age Ageing* **2022**, *51*, 1–9, doi:10.1093/ageing/afab237.

59. Chen, Y.; Lu, S.; Wu, Y.; Shen, Y.; Zhao, H.; Ding, S.; Feng, X.; Sun, L.; Tao, X.; Li, J.; et al. Change in Serum Level of Interleukin 6 and Delirium After Coronary Artery Bypass Graft. *Am. J. Crit. Care* **2019**, *28*, 462–470, doi:10.4037/ajcc2019976.
